# 4-(Phenylsulfanyl) Butan-2-One Attenuates the Inflammatory Response Induced by Amyloid-β Oligomers in Retinal Pigment Epithelium Cells

**DOI:** 10.3390/md19010001

**Published:** 2020-12-23

**Authors:** Peeraporn Varinthra, Shun-Ping Huang, Supin Chompoopong, Zhi-Hong Wen, Ingrid Y. Liu

**Affiliations:** 1 Institute of Medical Sciences, Tzu Chi University, Hualien 970, Taiwan; vpeeraporn@gms.tcu.edu.tw; 2 Department of Molecular Biology and Human Genetics, Tzu Chi University, Hualien 970, Taiwan; sphophdoc1688@gms.tcu.edu.tw; 3 Department of Anatomy, Faculty of Medicine Siriraj Hospital, Mahidol University, Bangkok 10700, Thailand; supin.cho@mahidol.ac.th; 4 Department of Marine Biotechnology and Resources, National Sun Yat-sen University, Kaohsiung 804, Taiwan; wzh@mail.nsysu.edu.tw

**Keywords:** coral, 4-(Phenylsulfanyl) Butan-2-One, inflammatory responses, amyloid-β, retinal pigment epithelium cells

## Abstract

Age-related macular degeneration (AMD) is a progressive eye disease that causes irreversible impairment of central vision, and effective treatment is not yet available. Extracellular accumulation of amyloid-beta (Aβ) in drusen that lie under the retinal pigment epithelium (RPE) has been reported as one of the early signs of AMD and was found in more than 60% of Alzheimer’s disease (AD) patients. Extracellular deposition of Aβ can induce the expression of inflammatory cytokines such as IL-1β, TNF-α, COX-2, and iNOS in RPE cells. Thus, finding a compound that can effectively reduce the inflammatory response may help the treatment of AMD. In this research, we investigated the anti-inflammatory effect of the coral-derived compound 4-(phenylsulfanyl) butan-2-one (4-PSB-2) on Aβ_1-42_ oligomer (oAβ_1-42_) added to the human adult retinal pigment epithelial cell line (ARPE-19). Our results demonstrated that 4-PSB-2 can decrease the elevated expressions of TNF-α, COX-2, and iNOS via NF-κB signaling in ARPE-19 cells treated with oAβ_1-42_ without causing any cytotoxicity or notable side effects. This study suggests that 4-PSB-2 is a promising drug candidate for attenuation of AMD.

## 1. Introduction

Age-related macular degeneration (AMD) is a degenerative macular disease that causes vision loss in the aged population [1]. It can be categorized into wet (neovascular AMD) and dry (atrophic AMD) AMD. The wet type is found in around 10–20% of AMD patients and results from abnormal growth of blood vessels. The breaking and leakage of blood vessels leads to irreversible damage to the macula and photoreceptors, in turn leading to vision loss. Blocking the activity of vascular endothelial growth factor (VEGF) is used as a treatment for wet-type AMD [2,3], whereas there is no approach yet for treating dry-type AMD. The dry-type AMD shows slower progression than wet-type AMD, and it affects approximately 80–90% of AMD patients. It is also associated with the formation of drusen [4]. Drusen are small yellow deposits in the macular area and are a common pathological hallmark of early AMD. Increased size and number of drusen contribute to a higher risk of AMD development and are related to retinal pigment epithelium (RPE) dysfunction, RPE atrophy, and photoreceptor degeneration [5,6]. The RPE is a monolayer of pigmented cells derived from the neuroectoderm and is located in between the neurosensory retina and the vascular choroid. The important functions of RPE cells are to maintain homeostasis of the outer retina, including helping the absorption of light, phagocytosis of old rod outer segments, transportation of nutrients and ions, protection from oxidative stress, immune privilege, and secretion of cytokines [7,8]. The amyloid-beta (Aβ) aggregates existing in drusen are related to increased secretion of inflammatory cytokines from RPE cells [9]. 

Aβ peptide contains 39–43 amino acids and presents mainly in senile amyloid plaques in the brain of Alzheimer’s disease (AD) patients and in the drusen of AMD patients [6,10,11]. In eyes, Aβ aggregation is primarily located among the outer segments of photoreceptors and between the RPE and Bruch’s membranes. oAβ_1-42_ secretion is elevated in human RPE cells by aging and is associated with oxidative stress [12], autophagy [13], and expressions of inflammatory molecules such as interleukin (IL)-1β, IL-6, tumor necrosis factor-α (TNF-α), cyclooxygenase-2 (COX-2), and inducible nitric oxide synthase (iNOS) [14,15]. These inflammatory-related cytokines and molecules were also increased in AMD patients [16,17,18]. Therefore, finding and developing a novel compound that can effectively reduce the inflammatory response in retinal cells is urgent for treating AMD. 

In the past decade, more than 20% of novel marine compounds have been discovered from soft corals [19]. The natural products isolated from soft corals have been demonstrated to exhibit various biological activities such as anti-tumor, anti-viral, and anti-inflammatory functions with minimum adverse effects [19,20,21,22,23]. The compound 4-(phenylsulfanyl) butan-2-one (4-PSB-2), modified from dihydroaustrasulfone alcohol, is synthetic precursor of soft coral (*Cladiella australis*)-derived natural compound, austrasulfone. It has an anti-melanogenic effect via the suppression of tyrosine kinase activity in zebrafish embryos [24]. It can also reduce expression levels of iNOS, and COX-2 increased after the optic nerves of rats were crushed [25]. Based on these lines of evidence, we investigated whether 4-PSB-2 can suppress inflammatory responses in human adult retinal pigment epithelial cell line (ARPE-19 cells) treated with oAβ_1-42_. Here, we report that 4-PSB-2 can effectively reduce the expressions of TNF-α, COX-2, and iNOS via NF-κB signaling in oAβ_1-42_-treated ARPE-19 cells without notable cytotoxicity. Our results suggest that 4-PSB-2 is a promising therapeutic compound for treating AMD.

## 2. Results

### 2.1. The Addition of oAβ_1-42_ Caused Morphological Changes and Mild Cell Death in ARPE-19 Cells

**Figure 1:**
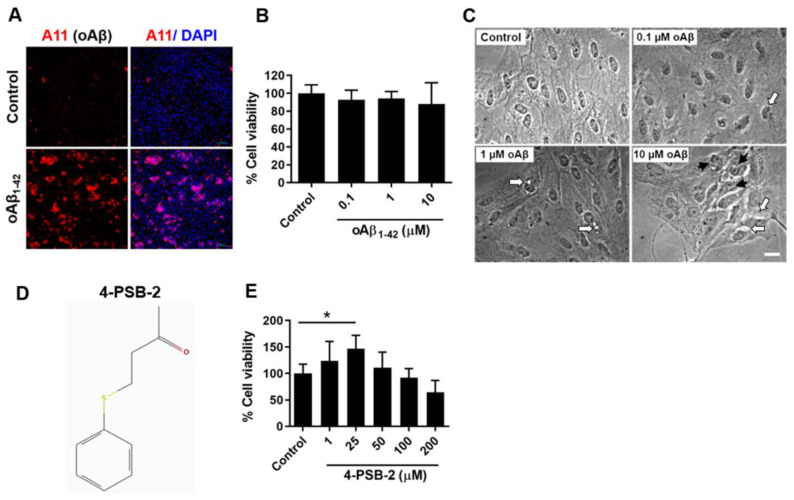
The effects of oAβ_1-42_ and 4-PSB-2 on the viability of human adult retinal pigment epithelial cell line (ARPE-19 cells). (**A**) The expression of oAβ_1-42_ in ARPE-19 cells: A11 (red), oAβ marker; DAPI (blue), nucleus; Bar, 100 µm. (**B**) A dose–response curve for RPE cells stimulated with 0.1 to 10 µM solutions of oAβ_1-42_ for 48 h demonstrates the mild effect of 10 µM oAβ_1-42_ on the viability of ARPE-19 cells (*n* = 5–6/group). (**C**) The morphology of ARPE-19 cells after 0.1 to 10 µM of oAβ_1-42_ treatment compared to control; Bar, 20 µm. The irregular shapes of cell bodies and nuclei of ARPE-19 cells (black arrows) and small vesicles in the cytoplasm (white arrows) were observed after oAβ_1-42_ treatment. (**D**) Chemical structure of 4-PSB-2. (**E**) ARPE-19 cells were treated with 0.1% dimethyl sulfoxide (DMSO) in control, 4-PSB-2 (1, 25, 50, 100, and 200 µM) for 24 h. MTT analysis showed that 4-PSB-2 was not toxic to ARPE-19 cells, and 25 µM of 4-PSB-2 significantly increased cell viability.(* indicates *p* ≤ 0.001 between the groups).

**Figure 2:**
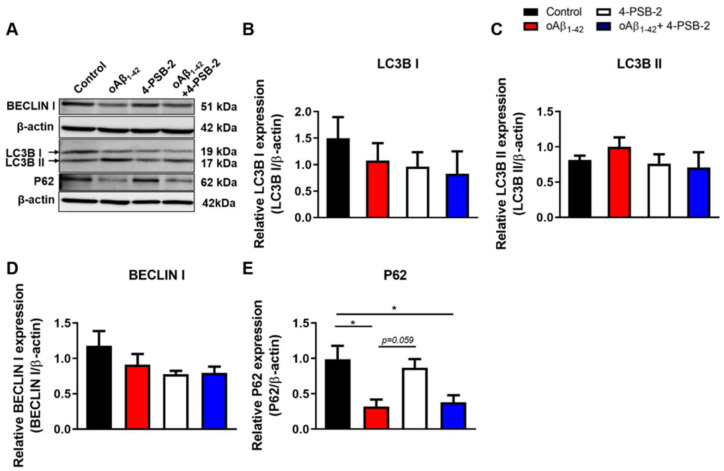
The effects of 4-PSB-2 on the autophagy markers expression in oAβ_1-42_-induced ARPE-19 cells. The experimental design is shown in Figure 3A. (**A**) The expressions of BECLIN 1, LC3B I, LC3B II, and p62 in ARPE-19 cells were assessed by Western blotting. (**B**–**E**) Quantitative analysis of the expressions of BECLIN 1, LC3B I, LC3B II, and p62 in ARPE-19 cells treated with the 4-PSB-2 and/or oAβ_1-42_. (**B**–**D**) The expression levels of BECLIN 1, LC3B I, and LC3B II were not significantly different between the groups after oAβ_1-42_ and/or 4-PSB-2 treatment. (**E**) The expression of p62 in the oAβ_1-42_ group was significantly decreased compared to the control group but was not rescued by 4-PSB-2 treatment. The results were plotted as means ± SEMs, * indicates *p* ≤ 0.05 between the groups.

RPE cells are the major cell type affected by oAβ_1-42_ in AMD [26]. To investigate the effect of adding oAβ_1-42_ in ARPE-19 cells, the Aβ peptide was incubated at 37 °C for 24 h, and the presence of the oligomeric form was further confirmed by immunocytochemistry staining of A11 or Aβ oligomer markers (Figure 1A). Three different concentrations of oAβ_1-42_ (0.1, 1, and 10 µM) were administered to ARPE-19 cells for 48 h. Then, the thiazolyl blue tetrazolium blue (MTT) assay was performed and showed no significant cell death (F(_3,30_) = 0.792, *p* = 0.513; Figure 1B). However, the morphological changes were observed in the 10 µM of oAβ_1-42_ group. Cell bodies and nuclei of ARPE-19 cells with added oAβ_1-42_ became smaller, and the cytoplasm contained many small vesicles (Figure 1C), which were suspected to be autophagosomes. Autophagy is a self-clearance mechanism that leads to the transport of cytoplasmic materials to vesicles for degradation and recycling. Autophagic dysfunction has been observed in AMD and is linked to the progression of disease [27,28]. To determine whether the small vesicles observed in the oAβ_1-42_-treated ARPE-19 cells were autophagosomes, we investigated the expression of autophagy-related molecules in oAβ_1-42_-treated ARPE-19 cells. Expressions of several autophagy-related molecules including LC3B I (Figure 2B), LC3B II (Figure 2C), BECLIN 1 (Figure 2D), and p62 (Figure 2E) were detected, indicating the existence of autophagasomes. p62 expression was decreased, suggesting an increase in autophagic activity.

### 2.2. 4-PSB-2 Increased Cell Viability in ARPE-19 Cells 

To examine the cytotoxic effect of 4-PSB-2 (Figure 1D) on ARPE-19 cells, we first applied five different concentrations of 4-PSB-2 (1, 25, 50, 100, and 200 µM) to ARPE-19 cell cultures for 24 h, and we used the MTT assay to measure cell viability. Results of the MTT analysis indicated that 4-PSB-2 did not cause toxicity in ARPE-19 cells; on the other hand, 25 µM of 4-PBS-2 significantly enhanced cell viability (F(_5,28_) = 6.818, *p* < 0.001; Figure 1E). 

### 2.3. 4-PSB-2 Repressed Elevated Expression of Inflammation Markers in oAβ_1-42_-Treated ARPE-19 Cells

**Figure 3:**
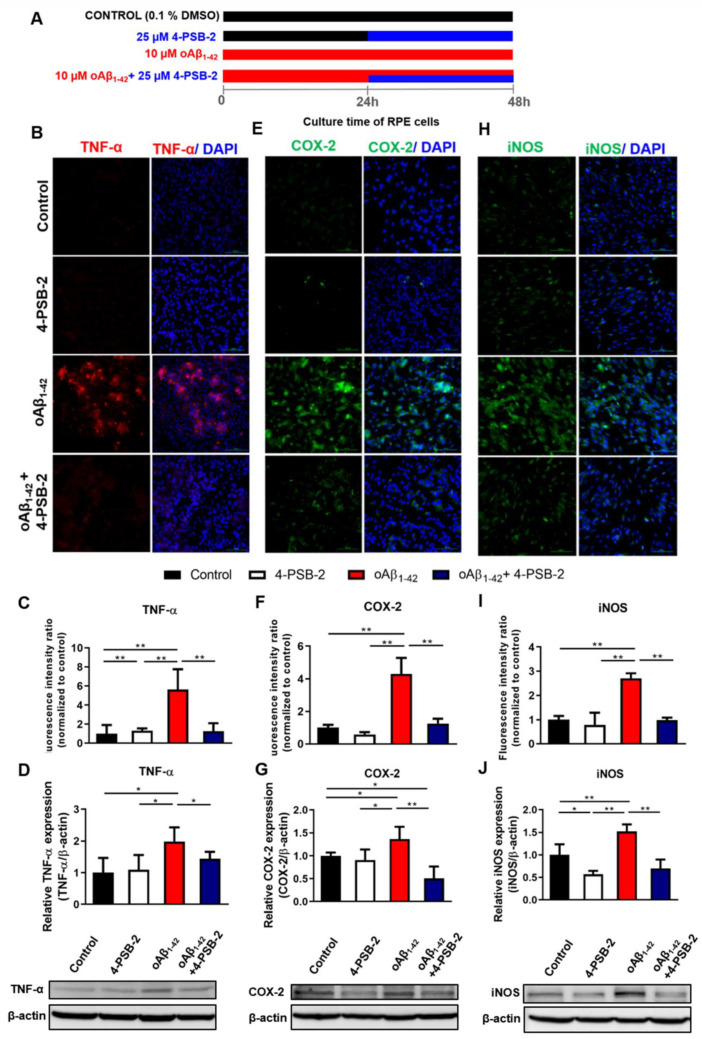
The effects of 4-PSB-2 on oAβ_1-42_-induced expression of inflammatory markers in ARPE-19 cells. (**A**) The illustrated protocols of ARPE-19 cells added with 4-PSB-2 and/or oAβ_1-42_. The ARPE-19 cells were cultured for a total of 48 h. For the control group, 0.1% dimethyl sulfoxide (DMSO) was added only. Twenty-five micromolars of 4-PSB-2 was added after the cells cultured for 24 h (from 24 h to 48 h) to test drug-only effects. To test the effects of 4-PSB-2 on the oAβ_1-42_-induced inflammatory response, in the cell cultures treated with 10 µM oAβ_1-42_, 25 µM of 4-PSB-2 was added from 24 h to 48 h. Fluorescence immunocytochemical staining and Western blot analysis of (**B**–**D**) TNF-α, (**E**–**G**) COX-2, and (**H**–**J**) iNOS expressions were significantly increased in oAβ_1-42_-treated ARPE-19 cells and decreased after 4-PSB-2 administration for 24 h. The results are plotted as means ± SEMs. * indicates *p* ≤ 0.05, and ** indicates *p* ≤ 0.001 between the groups. TNF-α (red), COX-2 and iNOS (green), and DAPI (blue) (nuclei), Bar = 100 µm.

4-PSB-2 has been reported to have anti-inflammatory and neuroprotective effects via inhibiting iNOS and COX-2 expression in a rat optic nerve crush model [25]. We thus wanted to investigate whether it had the same effects on oAβ_1-42_-treated ARPE-19 cells. MTT results are demonstrated in Figure 1E. ARPE-19 cells were administered with 10 µM of oAβ_1-42_, and 24 h later 4-PSB-2 was added, and cells were allowed to rest for an additional 24 h (Figure 3A). Then, the expression levels of inflammatory cytokines were measured. We detected higher expression levels of inflammatory cytokines including TNF-α, COX-2, and iNOS after 10 µM of oAβ_1-42_ administration. Notably, treatment with 4-PSB-2 significantly reduced the increased expression levels of TNF-α (F(_3,32_) = 28.767, *p* < 0.001; F(_3,16_) = 5.852, *p* < 0.01; Figure 3B–D), COX-2 (F(_3,32_) = 10.484, *p* < 0.001; F(_3,16_) = 7.192, *p* < 0.01; Figure 3E–G), and iNOS (F(_3,32_) = 9.977, *p* < 0.001; F(_3,12_) = 23.169, *p* < 0.001; Figure 3H–J). We also investigated the effect of 4-PSB-2 in oAβ_1-42_-treated ARPE-19 cells on the expression of autophagy-related molecules. The results indicated that 25 µM of 4-PSB-2 treatment did not significantly decrease the expression levels of autophagy-related molecules (F(_3,12_) = 0.641, *p* = 0.603, F(_3,12_) = 1.786, *p* = 0.203, F(_3,12_) = 0.750, *p* = 0.543, F(_3,12_) = 6.299, *p* < 0.01.; Figure 2B–E). 

### 2.4. 4-PSB-2 Attenuates Inflammatory Responses through NF-κB Signaling in ARPE-19 Cells

**Figure 4:**
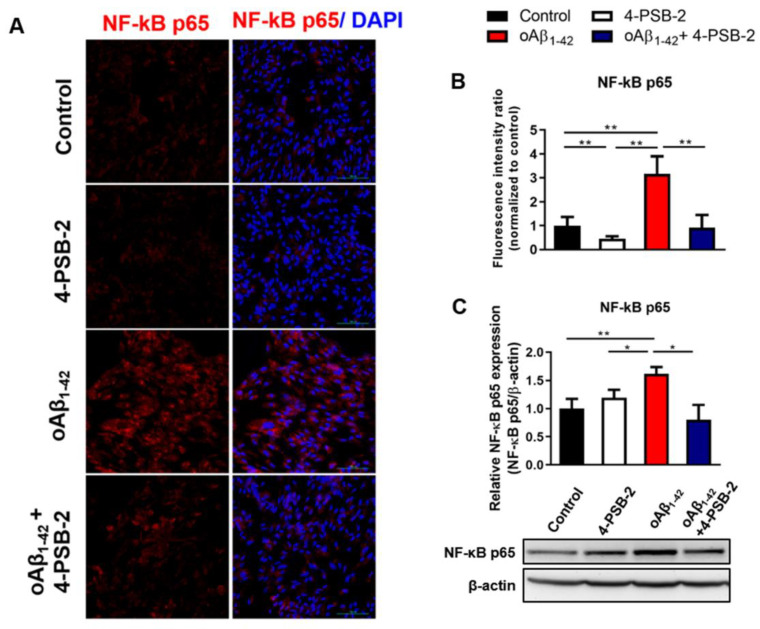
The effect of 4-PSB-2 on NF-κB signaling in ARPE-19 cells treated with oAβ_1-42_. (**A**,**B**) Fluorescence immunocytochemical staining and the quantitative results of NF-κB p65 expression in ARPE-19 cells were significantly enhanced after oAβ_1-42_ administration for 48 h. This phenomenon was suppressed with co-treatment of oAβ_1-42_ and 4-PSB-2. These results were reconfirmed with Western blot analysis (**C**). The results are plotted as means ± SEMs. * indicates *p* ≤ 0.05, and ** indicates *p* ≤ 0.001 between the groups. NF-κB p65 (red), and DAPI (blue) (nuclei); Bar, 100 µm.

NF-κB is a key mediator that regulates the inflammatory response through TNF-α activation [29]. It is also an upstream regulator of COX-2 and iNOS [30]. Next, we asked whether the anti-inflammatory effect of 4-PSB-2 on oAβ_1-42_-treated ARPE-19 cells was mediated by NF-κB signaling. The expression of NF-κB p65 was detected in oAβ_1-42_-treated ARPE-19 cells by immunocytochemical staining and Western blot analysis. oAβ_1-42_ treatment for 48 h significantly enhanced NF-κB p65 expression. Notably, treatment with 4-PSB-2 significantly inhibited the increased expression levels of NF-κB p65 (F(_3,32_) = 55.397, *p*< 0.001; F(_3,16_) = 4.607, *p* < 0.01; Figure 4A–C) induced by oAβ_1-42_.

## 3. Discussion

**Figure 5:**
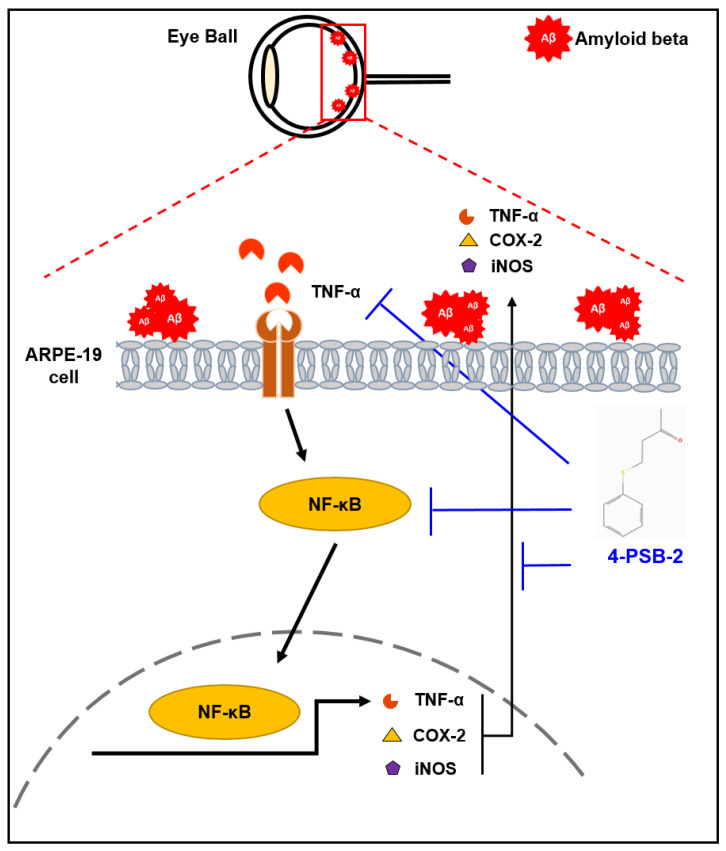
The effects of 4-PSB-2 in oAβ_1-42_-induced ARPE-19 cells. The extracellular deposition of oAβ_1-42_ increased expressions of TNF-α, COX-2, and iNOS in ARPE-19 cells via NF-κB signaling. Expressions of these inflammatory markers were suppressed by 4-PSB-2 treatment.

In the present study, we demonstrated that 4-PSB-2 has anti-inflammatory effects on oAβ_1-42_-treated ARPE-19 cells. The compound 4-PSB-2 can effectively suppress overexpression of TNF-α, COX-2, and iNOS via NF-κB signaling in ARPE-19 cells induced by oAβ_1-42_ treatment without causing any notable side effects (Figure 5).

Previous studies have reported that exposure of ARPE-19 cells to oAβ resulted in a reduction in cell viability in a dose- and time-dependent pattern [14,31]. Our results are in line with an earlier study showing that 10 µM of oAβ can alter the structure and function of RPE cells [5], but not cause cell death. It is noted that different concentrations of oAβ added to ARPE-19 cells cause distinct degrees of change. Five micromolars of oAβ increased ARPE-19 cell proliferation and inhibited apoptosis, whereas significant ARPE-19 cell death was observed after treatment with 25 µM of oAβ for 48 h [32]. Here, we found that the morphology of ARPE-19 cells was changed, with shrinking cell bodies and nuclei, and filling small vesicles in the cytoplasm. Transmission electron microscopy identified that the small vesicles were autolysosomes and autophagosomes [13]. Our results revealed that the p62-autophagy marker was decreased in ARPE-19 cells treated with oAβ peptides, which supports the findings from the aforementioned study. p62 directly binds to microtubule-associated protein 1A/1B-light chain 3 (LC3) to negatively modulate autophagic activity [13,33]. In addition, we found that expressions of inflammatory markers including TNF-α, COX-2, and iNOS were increased via NF-κB signaling in ARPE-19 cells induced with oAβ_1-42_, which were consistent with studies on human RPE cells exposed to oAβ_1-42_ [14,15] and AMD patients [16,17,18]. Interestingly, a study in the Russian population also reported a significant association between AMD and single-nucleotide polymorphisms of TNF-α [34]. TNF-α, a proinflammatory cytokine, is synthesized and secreted by activated macrophages and T-cells, and it regulates the biological activities of cells [35]. Chronic activation of TNF-α in RPE cells can change cell morphology, alter tight-junction organization, and decrease the immunosuppressive capacities by inducing transforming growth factor β (TGF-β) expression [36]. The activation of TNF-α is mediated by NF-κB, which is the upstream mediator of COX-2 and iNOS [29]. Inhibition of NF-κB signaling can reduce the inflammatory expressions and angiogenic factors in RPE cells induced by oAβ_1-42_ [37]. COX-2 is an enzyme inducible by pathologic stimuli such as lipopolysaccharides, IL-1β, TNF-α, and NF-κB [38]. Previous studies have shown that the expression of COX-2 in human choroidal neovascular membranes was related to AMD pathology by increasing the secretion of VEGF and TGF-β [38,39]. iNOS is an inducible isoform of nitric oxide and is induced by inflammatory cytokines. The increase in iNOS expression in choroidal neovascular membranes from patients with AMD directly links with VEGF [40]. Taken together, the overexpressions of inflammatory cytokines presented in ARPE-19 cells induced by oAβ_1-42_ may be one of the causal factors of AMD; thus, reduction in the inflammatory response may become an effective therapeutic approach for this disease.

The compound 4-PSB-2, modified from dihydroaustrasulfone alcohol, is a synthetic precursor of *Cladiella australis*-derived natural compound, austrasulfone. Both austrasulfone and dihydroaustrasulfone alcohol are anti-inflammatory compounds that can inhibit the expressions of iNOS and COX-2 proteins in LPS-stimulated RAW264.7 macrophage cells [22]. Besides, the chemical structure of 4-PSB-2 is similar to BAY 11-7082, which also shows anti-inflammatory effects via NF-κB signaling [41]. Thus, the two compounds probably repress the inflammatory response via a similar mechanism. However, 25 µM of 4-PSB-2 did not modulate autophagic activity. Either 25 µM is not an effective dosage to rescue autophagic dysfunction, or this compound does not affect the autophagy pathway. Our results suggest that 4-PSB-2 may be a promising drug candidate for treating AMD. Further time-course, pharmacokinetic, and pharmacological metabolism studies are necessary to explore the possibility of further clinical application.

## 4. Materials and Methods 

### 4.1. Cell Culture

A human retinal pigment epithelial cell line (ARPE-19) was provided by Rong-Kung Tsai at Institute of Medical Sciences, Tzu Chi University (Taiwan), and cultured in Dulbecco’s modified Eagle’s medium/nutrient mixture F-12 (DMEM/F12), containing 10% fetal bovine serum (FBS), 100 U/mL penicillin, and 100 µg/mL streptomycin at 37 °C in 5% CO_2_ and 95% air. All cell culture reagents were obtained from Thermo Fisher Scientific (Waltham, MA, USA). The cells were seeded in 24-well plates (1 × 10^5^ cells/well) on 12 × 12 mm^2^ pieces of glass for immunocytochemical staining, 6-well plates (7 × 10^5^ cells/well) for protein collection, and 96-well plates (2 × 10^4^ cells/well) for cell viability assays. 

### 4.2. Preparation of Oligomeric Aβ_1-42_ Solution

The Aβ_1-42_ peptide (Bacham, Merseyside, UK) was dissolved in 0.1% NH_4_OH to a final concentration of 1 mg/mL. The Aβ_1-42_ peptide was incubated at 37 °C for 24 h for aggregation, which was verified by Western blotting and immunocytochemical staining (Figure 1A). The Aβ_1-42_ peptide was dissolved in medium to final concentrations of 0.1, 1, and 10 µM for cytotoxicity examination. 

### 4.3. Preparation of 4-PSB-2 Solution and Treatment 

In this study, 4-PSB-2 was provided by the Research Center of National Research Program for Biopharmaceuticals, Taiwan, and its structure is shown in Figure 1D. To verify the cytotoxicity of 4-PSB-2, it was dissolved in DMSO and medium to final concentrations of 1, 25, 50, 100, and 200 µM. 

### 4.4. Cell Viability Assay

ARPE-19 cells were plated in 96-well plates containing 10% FBS DMEM/F12 medium and cultured for 24 h. ARPE-19 cell cytotoxicity was measured at oAβ_1-42_ doses of 0.1, 1, and 10 µM and 4-PSB-2 doses of 1, 25, 50, 100, and 200 µM. Cell viability was measured by thiazolyl blue tetrazolium blue (MTT; Sigma-Aldrich, St. Louis, MO, USA). Briefly, 10 µL of MTT solution (5 mg/mL) were added to each well and incubated for 3 h at 37 °C. After removing the supernatant, 100 µL DMSO was added into each well. The intensity was measured colorimetrically at 570 nm with a microplate reader (Thermo Scientific Multiskan Spectrum, USA).

### 4.5. Western Blot Analysis

ARPE-19 cells were collected from 6-well plates and then homogenized in an ice-cold radioimmunoprecipitation assay (RIPA) lysis buffer containing phosphatase and protease inhibitors (F. Hoffmann-La Roche AG, Basel, Switzerland). The samples were sonicated and centrifuged for 15 min at 13,500× *g* at 4 °C. The supernatants were collected, and the protein concentration was measured with Bradford protein assay (Bio-Rad Laboratories, Hercules, CA, USA). Equal amounts of proteins from ARPE-19 cells were separated by 12% sodium dodecyl sulfate–polyacrylamide gel electrophoresis (SDS-PAGE) and transferred to nitrocellulose membranes. The membranes were blocked with 1% bovine serum albumin (BSA) for 1 h at room temperature and incubated overnight at 4 °C with the following primary antibodies: rabbit anti-TNF-alpha (1:1000, Abcam, Cambridge, UK), goat anti-COX-2 (1:500, Santa Cruz Biotechnology, Dallas, TX, USA), rabbit anti-iNOS (1:500, Thermo Fisher Scientific), rabbit anti-NF-κB p65 (1:1000, Santa Cruz Biotechnology), rabbit anti-BECLIN I (1:200, Abcam), rabbit anti-LC3B I/II (1:200, Abcam), rabbit anti-p62 (1:200, Abcam), and mouse anti-β-actin (1:10,000, Sigma-Aldrich). After that, the membranes were washed three times with 1X phosphate-buffered saline (PBS) containing 0.1% Tween-20 and incubated with the corresponding conjugated antibodies, including a horseradish peroxidase-conjugated (HRP) anti-mouse antibody (1:10,000, Invitrogen, Carlsbad, CA, USA), HRP-conjugated goat anti-rabbit or anti-mouse antibody (1:10,000, Invitrogen), and HRP-conjugated donkey anti-goat antibody (1:10,000, Invitrogen), for 1 h at room temperature. The proteins of specific molecular weights were visualized using enhanced chemiluminescence reagents (Western Lightning^®^ Plus-ECL, PerkinElmer, MA, USA) and detected by a UVP BioSpectrum 810 imaging system. Band intensity was quantified using ImageJ (downloaded from National Institutes of Health, Bethesda, MD, USA).

### 4.6. Immunocytochemical Staining and Image Analysis

ARPE-19 cells were seeded on coverslips overnight. After oAβ_1-42_ and/or 4-PSB-2 treatment for 24 h, the cells were fixed with 4% paraformaldehyde and blocked with 2% bovine serum albumin. Then, the cells were incubated overnight at 4 °C with the following primary antibodies: rabbit anti-Aβ oligomer (A11) (1:500, Thermo Fisher Scientific), rabbit anti-TNF-α (1:300, Abcam), goat anti-COX-2 (1:500, Santa Cruz Biotechnology), rabbit anti-iNOS (1:300, Thermo Fisher Scientific), and rabbit anti-NF-κB p65 (1:300, Santa Cruz Biotechnology). Then, they were incubated at room temperature for 1 h with the following secondary antibodies: Alexa 594- or Alexa 488-conjugated goat anti-rabbit IgG (1:300, Thermo Fisher Scientific) and Alexa 488-conjugated donkey anti-goat IgG (1:300, Thermo Fisher Scientific). After washing with PBS, the cells were counterstained with DAPI for 5 min, mounted with Fluoromount™ aqueous mounting medium, and observed under a fluorescence microscope (Nikon ECLIPSE Ni-E, Tokyo, Japan). For calculating the positive area, the percentage of each antibody (450 µm × 450 µm) was quantified using ImageJ software.

### 4.7. Statistical Analysis

The mean ± standard error of the mean (mean ± SEM) were calculated and plotted. The data from the cell viability assay were analyzed by a two-tailed Mann–Whitney test. Western blotting and immunocytochemical staining data were analyzed by one-way ANOVA. Statistical significance for the differences among the groups was established at a *p*-value < 0.05. All graphs were plotted with GraphPad Prism 8.0 software (San Diego, CA, USA). 

## 5. Conclusions

In this study, we identified that 4-PSB-2 exhibits anti-inflammatory effects via NF-κB signaling in oAβ_1-42_-treated ARPE-19 cells without notable side effects. Our results suggest a novel therapeutic approach to AMD.
